# Effects of propranolol on fear of dental extraction: study protocol for a randomized controlled trial

**DOI:** 10.1186/s13063-015-1065-7

**Published:** 2015-11-25

**Authors:** Serge A. Steenen, Arjen J. van Wijk, Roos van Westrhenen, Jan de Lange, Ad de Jongh

**Affiliations:** Department of Oral and Maxillofacial Surgery, Academic Medical Center of the University of Amsterdam, Meibergdreef 9, 1105 AZ Amsterdam, The Netherlands; Department of Social Dentistry and Behavioral Sciences, Academic Centre for Dentistry Amsterdam, Gustav Mahlerlaan 3004, 1081 LA Amsterdam, The Netherlands; Department of Psychiatry, Erasmus University Medical Center, ‘s-Gravendijkwal 230, 3015 CE Rotterdam, The Netherlands; School of Health Sciences, Salford University, Salford, UK

**Keywords:** Dental anxiety, Dental extraction, Tooth extraction, Third molar, Propranolol, Randomized controlled trial

## Abstract

**Background:**

Undergoing an extraction has been shown to pose a significantly increased risk for the development of chronic apprehension for dental surgical procedures, disproportionate forms of dental anxiety (that is, dental phobia), and symptoms of post-traumatic stress. Evidence suggests that intrusive emotional memories of these events both induce and maintain these forms of anxiety. Addressing these problems effectively requires an intervention that durably reduces both the intrusiveness of key fear-related memories and state anxiety during surgery. Moreover, evidence suggests that propranolol is capable of inhibiting “memory reconsolidation” (that is, it blocks the process of storing a recently retrieved fear memory). Hence, the purpose of this trial is to determine the anxiolytic and fear memory reconsolidation inhibiting effects of the ß-adrenoreceptor antagonist propranolol on patients with high levels of fear in anticipation of a dental extraction.

**Methods/Design:**

This trial is designed as a multicenter, randomized, placebo-controlled, two-group, parallel, double-blind trial of 34 participants. Consecutive patients who have been referred by their dentist to the departments of oral and maxillofacial surgery of a University hospital or a secondary referral hospital in the Netherlands for at least two tooth and/or molar removals and with self-reported high to extreme fear in anticipation of a dental extraction will be recruited. The intervention is the administration of two 40 mg propranolol capsules 1 hour prior to a dental extraction, followed by one 40 mg capsule directly postoperatively. Placebo capsules will be used as a comparator. The primary outcome will be dental trait anxiety score reduction from baseline to 4-weeks follow-up. The secondary outcomes will be self-reported anxiety during surgery, physiological parameters (heart rate and blood pressure) during recall of the crucial fear-related memory, self-reported vividness, and emotional charge of the crucial fear-related memory.

**Discussion:**

This randomized trial is the first to test the efficacy of 120 mg of perioperative propranolol versus placebo in reducing short-term (“state”) anxiety during dental extraction, fear memory reconsolidation, and lasting dental (“trait”) anxiety in a clinical population. If the results show a reduction in anxiety, this would offer support for routinely prescribing propranolol in patients who are fearful of undergoing dental extractions.

**Trial registration:**

ClinicalTrials.gov identifier: NCT02268357, registered on 7 October 2014. The Netherlands National Trial Register identifier: NTR5364, registered on 16 August 2015.

## Background

### Epidemiology and current management of fear for dental extractions

Tooth or molar removals are among the most strongly feared procedures in Dentistry and Oral and Maxillofacial Surgery [1, 2]. In the United States, approximately 55 million extractions and surgical removals are carried out each year [3]. In the majority of cases, having a tooth removed causes immediate but generally transitory emotional distress [4–7]. This type of transitory (“state”) anxiety not only produces discomfort but can also induce patient behavior that impedes surgery, thereby increasing operative time and complicating postoperative recovery [8, 9]. Hence, to be capable of effectively performing dental treatment, Oral and Maxillofacial surgeons and specialized dental fear clinics commonly employ oral and intravenous sedatives such as benzodiazepines and nitrous oxide. However, the application of conscious sedation requires thorough training, and sedation-related incidents are common [10]. A publication by the National Health Service of the United Kingdom showed that, in the period between 2004 to 2008, 1,529 incidents were reported regarding conscious sedation with midazolam; in three cases, the patient died [10]. Further disadvantages of these agents include reduced cognitive and motor function, and anterograde amnesia, which may interfere with long-term anxiety reduction [11, 12]. Moreover, to date, only limited evidence exists for the effectiveness of these agents at reducing dental anxiety [13]. Because anxiety treatment failure rates of 88 to 91 % for intravenous sedation and 61-70 % for nitrous oxide have been found at 1-year follow-up after treatment [14], new safe, easily applied and cost-effective interventions for use within the dental and oral surgical setting are warranted.

### Role of traumatic dental experiences in the etiology and maintenance of dental anxiety

Having undergone an extraction had been found to pose an increased risk for developing persistent anxiety [6, 7, 15]. A small portion of approximately 4 to 8 % of patients suffer from elevated dental trait anxiety or even post-traumatic stress disorder (PTSD) symptoms 1 month after surgical removal of their wisdom teeth [6, 7]. This suggests that the psychological impact of a dental extraction may not be limited to temporary distress but also that a dental extraction is a potential psychologically traumatic experience to at least some patients.

A wide array of studies have shown that dental fear and anxiety are deeply rooted in the experience of previous negative dental or other types of distressing and traumatic life events, for example, [15–18]. A recent case-control study (total n = 153) among dental phobic individuals, subthreshold dental phobics and normal controls, showed that apprehension of dental treatment is strongly associated with the presence of memories of earlier distressing events [19]. For example, 97 % of subthreshold (non)phobic individuals have reported a memory of an aversive event, predominantly in the dental setting, that initiated or exacerbated their dental anxiety; this was significantly higher than the proportion of normal controls reporting such a memory [19]. Emotional disturbance, vividness and sense of reliving of these memories were significantly higher in the subthreshold phobic group and dental phobic group than in the normal controls [19]. Notably, normal controls reported lower levels of intrusiveness and avoidance scores (features typically seen in individuals suffering from PTSD) than both phobic and subthreshold phobic groups (all p values < 0.001). This suggests that the memories of the previous distressing dental events that initiated the dental fear play an important role in the maintenance of the individual’s current anxiety and fear response [19]. This is of importance, as research has indicated that dental anxiety is likely to lead to avoidance of dental care with subsequent adverse effects on oral health status and quality of life [20].

### Rationale for testing the effects of perioperative propranolol on dental anxiety

Treating apprehension or fear of dental treatment effectively requires an intervention that reduces the (1) state anxiety during the surgery, (2) the emotional charge of key fear-related memories, and (3) patients’ level of dental trait anxiety. There are indications that the ß-blocker propranolol is capable of bringing about these anxiolytic effects.

Propranolol, a ß_1,2_-adrenoreceptor antagonist, competes at the receptor level with catecholamines, thereby blocking their orthosympathetic effects [21]. Clinically, propranolol is used widely to target peripheral sites of the noradrenergic system to treat hypertension [22], coronary artery disease [23] and tachyarrhythmias [24]. In addition, propranolol can be deployed to block ß-adrenoreceptors in the central nervous system, as the lipophilic compound readily enters the blood-brain-barrier.

Several trials studying off-label use of propranolol have been conducted, such as its application in the treatment of high levels of trait anxiety [25–27], substance disorder and withdrawal symptoms [28], schizophrenia [29], autism [30], and aggression [31]. In addition, propranolol has been shown to mitigate milder distressing states such as exam nerves [32–34], stage fright [35], performance anxiety among musicians [36] and surgeons [37], and fear of undergoing surgery [38–40]. Evidence suggests that propranolol positively influences dental state anxiety and reduces the storage of fear memories.

### Evidence for effects of propranolol on dental state anxiety

Currently one randomized, controlled clinical trial (RCT) evaluating the state anxiolytic effects of propranolol in dental phobia has been published. It showed that, as compared to placebo, 80 to 120 mg of oral propranolol significantly diminished self-reported state anxiety during injection of local dental anesthesia (self-reported anxiety about the injection: 5.5 (2.75) versus 7.45 (2.0), p = .033, one-tailed) [41]. A limitation to this study is that follow-up data were lacking. This means that to date, the long-term effects of propranolol on dental trait anxiety are unknown.

### Evidence for effects of propranolol on fear memory storage

Because traumatic memories seem to play a key role in the maintenance and severity of dental trait anxiety [19], propranolol has potential for the treatment of this condition. Preclinical studies have shown that propranolol has the capacity to selectively block the protein synthesis required for the storage of aversive (emotional) memory while sparing declarative memory (factual memory) [42–50]. A recent meta-analysis of eight experiments with healthy human volunteers (total n = 308) supports this line of reasoning, as it showed that, compared to placebo, propranolol administered before memory reactivation reduces the expression of cue-elicited fear responses [51]. To date however, there is only one clinical trial supporting the effects of propranolol on the reconsolidation of traumatic memories. In this study, with chronic PTSD patients (n = 19), it was found that 40 mg of short-acting oral propranolol given prior to imaginary exposure to the disturbing memory of the event, followed by 60 mg of long-acting oral propranolol, significantly reduced physiologic responding to the memory one week later [52]. Although preclinical evidence is promising, clinical evidence is scarce. Therefore, the reconsolidation blocking capacities of propranolol need further research attention.

### Primary aim

The primary aim of this study is to determine whether administration of the active substance (two 40 mg propranolol capsules 1 hour prior to dental extraction, followed by one 40 mg capsule immediately postoperatively) results in a significantly greater reduction of dental trait anxiety in patients with self-reported high to extreme fear in anticipation of dental extraction, compared to the effects of the placebo comparator, from baseline to 4-week follow-up appointment.

### Secondary aims

The secondary aims are to determine whether the use of propranolol in patients with high self-reported levels of fear in anticipation of tooth or molar removal results in the following:

1. a significantly greater reduction of self-reported intraoperative (state) anxiety, compared to the placebo comparator, from baseline to the 4-week follow-up appointment;
2. a significantly greater decrease of physiological responding during recall of the crucial fear-related memory, compared to the placebo comparator, from baseline to the 4-week follow-up appointment;
3. a significantly greater decrease in specific phobia diagnoses, compared to the placebo comparator, from baseline to the 4-week follow-up appointment;
4. a significantly greater reduction of self-reported vividness and emotional charge of the crucial fear-related memory scores, compared to the placebo comparator, from the baseline to the 4-week follow-up appointment.

## Methods/Design

### Trial design

The trial is designed as a multicenter, randomized, placebo-controlled, two-group, parallel, double-blind superiority trial of 34 participants with an allocation ratio of 1:1.

### Study population

Adult participants of all genders will be recruited from the waiting list of patients referred to the departments of Oral and Maxillofacial Surgery of the Academic Medical Center of the University of Amsterdam (AMC), the Netherlands, and the department of Oral and Maxillofacial Surgery of the Spaarne Gasthuis Hospital in Haarlem, the Netherlands, by their dentists. Participants have an indication for the removal of at least two teeth and/or molars, and indicate finding a tooth or molar removal highly or extremely anxiety provoking.

### Eligibility criteria

#### Inclusion criteria

The inclusion criteria are as follows:

1. Signed written informed consent
2. Minimum age of 18 years on entry to the study
3. Self-reported high to extreme fear of tooth or molar removal
4. Dutch or English-speaking

#### Exclusion criteria

The exclusion criteria include the following:

1. Asthma or other obstructive pulmonary disease
2. Cardiac failure
3. Cardiac arrhythmia
4. Renal failure
5. Insulin-dependent diabetes mellitus
6. Pregnant or breast-feeding
7. Current use of another ß-adrenoreceptor antagonist
8. Current use of anxyiolytic or antidepressant medication
9. Currently in psychotherapy for dental anxiety
10. Systolic blood pressure < 100 mmHg

### Sample size calculation

A power analysis was performed for the primary outcome variable, dental trait anxiety as indexed by the Short-version of the Dental Anxiety Inventory (S-DAI). In order to detect a difference between the propranolol and control condition on changes in dental anxiety scores over time, a two-way (one-within and one-between subjects factor) repeated measures ANOVA will be used. Using G*Power 3.0 software, assuming a correlation of 0.50 between two repeated measurements, a medium size treatment effect (f = 0.25), a power of 0.80, an α significance level of 0.05, and two treatment conditions, the power analysis results in a total sample size of 34 persons.

### Study procedures

**Fig. 1:**
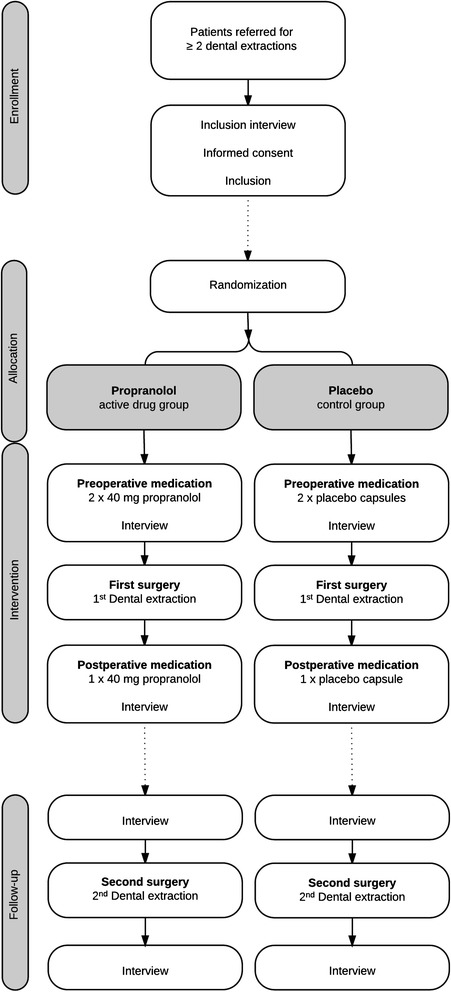
Flow-chart of study procedures

The study procedures and participant flow through each stage are summarized in a flow-chart (Fig. 1).

#### Screening and recruitment

Potential participants will be recruited from the waiting list of consecutive patients referred to the Department of Oral and Maxillofacial Surgery of the Academic Medical Center of the University of Amsterdam, the Netherlands, and the Department of Oral and Maxillofacial Surgery of the Spaarne Gasthuis Hospital in Haarlem, the Netherlands, by their dentists. The patients have an indication for the removal of at least two teeth and/or molars. They will be contacted by the investigators by telephone, and informed about the trial. After a short introduction, they will be asked to rate how anxiety provoking a tooth or molar removal is to them on a four-point scale (translated from Dutch); “not anxiety provoking at all” (1), “more or less anxiety provoking” (2), “fairly/highly anxiety provoking” (3), or “extremely anxiety provoking” (4). When patients indicate that a score of 3 or 4 applies to them, they will be asked whether they are interested in receiving a potential participant information letter at their home address. The patients will be offered the opportunity to contact an independent physician for questions regarding their participation. No financial or other form of compensation is provided for participation.

#### Informed consent

After patients have had sufficient time to reflect and decide to give written informed consent, they will be registered as participants in the trial.

#### First preoperative assessment

Participating patients will be asked to arrive at the waiting room of both outpatient Oral and Maxillofacial surgery clinics, 1 hour and 15 minutes prior to their appointment for ingestion of study medication, for a diagnostic interview and filling out questionnaires. Demographic variables (age, sex, highest level of education marital status, and country of birth) will be recorded.

Next, the presence of specific (that is, dental phobia in accordance with the DMS-IV TR criteria updated in 2000 [53] will be assessed using *the Phobia Checklist* containing four questions based on the DSM-IV TR criteria, developed for the assessment of dental phobia [54]. The Phobia Checklist contains four questions based on the Diagnostic and Statistical Manual of Mental disorders, text revision (DSM-IV TR) [53] criteria for specific phobia, developed for the assessment of dental phobia [54]. The Phobia Checklist has been validated and proven to be a valid diagnostic tool for this purpose (sensitivity = 0.95, specificity = 0.99, and an overall hit rate of 97 %). The presence of specific (that is, dental) phobia in accordance with the DSM 5 criteria updated in 2013 [55] will be indexed using an adapted version of *The Mini International Neuropsychiatric Interview Plus, Dutch version 5.0.0 section “Specific phobia” (H). The MINI-Plus* [56] is a structured diagnostic interview to systematically assess DSM-IV and 10^th^ revision of the International Classification of Diseases and Related Health Problems (ICD-10) diagnoses. A recent version of the MINI-Plus is not available; therefore, an adapted version will be used in which the new DSM 5 criteria [55] are addressed. Since only the presence of a specific phobia is of interest, only the section “Specific phobia” (H) will be used to assess the dichotomous specific phobia outcome.

To identify the memories of distressing events that initiated or exacerbated dental anxiety, the *Full Intrusions Interview,* a modified semi-structured interview adapted from Reynold and Brewin (1999) [57] will be used. Patients will be asked to report any spontaneous autobiographical memory that kept coming to mind over the previous week from a past event. If the last week had been exceptional, they will be prompted to think back to a typical week, or a week shortly before a dental appointment. The participant will be requested to identify the most disturbing memory. Based on this memory, the investigator will compose and record an approximately 30-second personal memory script, portraying the experience. This script of the most crucial traumatic memory is called M1. Characteristics of this memory will be subsequently determined (that is, avoidance and aversiveness propensity, the sense of here and now, the impact of and burden caused by the memory), after which patients will be requested to rate these characteristics on standardized continuous self-report VASs ranging from 0 to 100.

#### Treatment allocation

After the interview, patients will be randomized and allocated to receive either perioperative oral propranolol capsules or placebo capsules. Both groups will undergo two regular dental extraction procedures, with a 4-week interval.

#### First dental extraction procedure

One hour and 15 minutes before the surgery, participants will be approached by the investigator. They will then receive the first two capsules of study medication. The intervention group will receive 80 mg of oral propranolol (2 capsules) 1 hour and 15 minutes prior to treatment, and 40 mg of oral propranolol (1 capsule) directly after treatment. The control group will receive oral placebo capsules in the same order. The following hour is reserved to allow time for achieving an optimal plasma concentration of propranolol.

In the period between ingestion of the study medication and the surgery, a 30-minute screening by an investigator will take place (see paragraph: “Study outcome measures”). All outcomes will be registered on a case report form (CRF). After the interview, the patients will be guided back to the waiting room by the investigator. Next, they will be invited into the operating room by their surgeon. The surgeon will be either a senior staff member or a resident in Oral and Maxillofacial Surgery. During this first procedure, a prilocaine 3 % with felypressin (1:850,000) solution will be administered as local intraoral anesthetic to prevent a possible unwanted α-pressor reaction; a rare and potentially harmful pharmacological interaction of ß-adrenoreceptor antagonists with adrenaline that may result from the intravascular injection of the local anesthetic, causing bradycardia and hypertension.

In case of patients with a high level of fear of dental extractions, the surgeon will be notified that the patient has a “high or extreme” fear of dental extractions (the level of fear is not specified further) and is instructed to treat the patients accordingly.

#### First postoperative assessment

At completion of the surgical procedure, the dental surgeon will fill out the CRF containing the operative variables. Before leaving the clinic, the patient will again be shortly interviewed by the investigator. Only after the first dental extraction procedure will another semi-structured interview be conducted. This is meant to construct an approximately 30-second personal memory script portraying this first dental extraction procedure that the participant has just experienced (M2). The participant will subsequently receive one final capsule containing 40 mg of propranolol or placebo. The participant will be asked to fill in a small “intrusions diary” at home.

#### Second dental extraction procedure

At the 1-month follow-up, a second dental extraction at the hospital will be performed, this time without the study medication. Administration of all questionnaires before and after the procedure will be repeated, but this time, no full-intrusions interview will be conducted. During the second procedure, a regularly used local anesthetic solution of articaine 4 % with adrenaline (1:200,000) will be administered. At completion, the surgeon will fill out another CRF, and the participant will be asked to keep another “intrusions diary”.

#### Methods to maximize participant adherence to the intervention and follow-up procedures

The day before the first and second treatment, participating patients will receive a text message on their cell phone and an email at their home address reminding them of the appointments. The day after the first and second treatment, participants will receive another text message or email reminding them to fill out the “intrusions diary”.

### Investigational products

#### Active substance

The active medicinal product investigated is Propranolol HCl (a lipophilic ß_1,2_-adrenoreceptor antagonist) 3 x 40 mg tablets overencapsulated in three blue-white, size 00, oral capsules, complemented with microcrystalline cellulose PH 102.

The preoperative dose of 80 mg propranolol is intended to mitigate perioperative state anxiety. Previous research has shown that a dose of 40 mg of oral propranolol was inadequate to reduce the acute hyperadrenergic anxiety states in recently psychologically traumatized individuals [58], and that administration of 80 to 120 mg of oral propranolol significantly reduced self-reported anxiety during injection of local intraoral anesthesia in patients with dental phobia [41].

The administration of 40 mg of propranolol immediately postoperatively is targeted at blocking the reconsolidation process of emotional memory. Research suggests that a “window of opportunity” for ß-adrenergic modulation of memory reconsolidation exists for some hours after retrieval of a fear-related memory [50, 59]. Because propranolol has a half-life of approximately 4 hours, the initial preoperative dose may not exert sufficient ß-adrenergic receptor antagonizing effects during the full reconsolidation period.

#### Placebo comparator

The placebo comparators are three capsules, visually identical to the active substance, filled with pharmacologically inactive microcrystalline cellulose PH 102 as a sole ingredient.

### Sequence generation, allocation concealment and implementation

The random allocation sequence will be computer-generated. The allocation list is available only to the GMP-certified Hospital Pharmacy, where the study medication was produced and labeled, and to the Hospital Pharmacies of both study locations, where the study medication is conserved and provided. The manufacturer of the investigational medicinal products (GMP-certified Hospital Pharmacy) will randomize the allocation sequence; bottles consecutively issued by the study pharmacies will therefore contain either propranolol or placebo capsules in a random order.

### Blinding

Trial participants, investigators, data collectors, the statistician, and outcome assessors are blinded to the study-group assignments. Only the producing and providing hospital pharmacy staff are not blinded; the allocation of a participant will be unmasked before the end of the trial only in case of a serious adverse event related to the study medication.

### Study outcome measures

Dependent variables were derived from the questionnaires listed below (see Tables 1 and 2).

**Table 1:** Primary and secondary study outcome measures

		Measures			
		Primary outcome	Secondary outcomes		
		Trait anxiety (S-DAI)	State anxiety (VAS score)	Vividness/emotional charge (M1) propensity (VAS scores)	Physiological response (HR/BP)
Screening (t = 0)	Screening interview				
Intervention (t = 1)	Preoperative interview	X		X	X
	Postoperative interview		X		
Follow-up (t = 2)	Preoperative interview	X		X	X
	Postoperative interview		X		

*Note:* *BP* blood pressure, *HR* heart rate, *M1* crucial fear-related memory, *S-DAI* Short version of the Dental Anxiety Inventory, *VAS* visual analog scale

**Table 2:** Tertiary outcome measures

		Measures							
		Operative variables/qualitative aspect of the surgical experience (VASs)	Test of blind	IES	FCDS	Qualitative aspect of memory M2 (VASs)	Qualitative aspect of memory M1 (VASs)	Intrusions diary	Specific phobia diagnosis
Screening (t = 0)	Screening interview				X				X
Intervention (t = 1)	Preoperative interview			X			X		
	Postoperative interview	X	X			X		X	
Follow-up (t = 2)	Preoperative interview			X	X	X	X		X
	Postoperative interview	X						X	

*Notes:* *FCDS* fear of stimuli compromising the dental setting, *IES* Impact of Event Scale - Revised, *M1* crucial fear-related memory, *M2* memory of recent dental extraction procedure, *VAS* visual analog scale

#### Primary outcome measure

The primary aim is to assess the reduction in the dental trait anxiety score in the propranolol versus the placebo control group (Table 1). The change from baseline to 1-month follow-up in dental trait anxiety will be indexed with the Dutch version of the *Short version of the Dental Anxiety Inventory* (S-DAI) [60]. The items (for example, “when I know the dentist/oral-maxillofacial surgeon is going to extract a tooth, I am already afraid in the waiting room”) are answered on a five-point Likert-type scale, ranging from “totally not applicable to me” (1) to “totally applicable to me” (5). Total scores on this questionnaire range from 9 to 45. The clinical cut-off score for a patient to apply for treatment in a specialized dental fear clinic is a minimum of 28 points on the S-DAI [61].

#### Secondary outcome measures

The self-reported state anxiety during dental treatment from baseline to 1-month follow-up will be indexed using a visual analog scale (Table 1). Patients are asked to rate the question “How much anxiety did you experience during the last treatment” using a standardized continuous self-report VAS ranging from “no anxiety at all” 0 to “extreme anxiety” 100.

Change from baseline to 1-month follow-up in self-reported vividness and emotional charge of the crucial fear-related memory will be measured using visual analog scales that assess key characteristics of the traumatic memory that initiated or exacerbated the dental anxiety (M1) (Table 1). Patients will be asked to rate the questions “how vivid or detailed is the memory, if you bring up the memory now” and “how much emotion does the memory evoke, if you bring up the memory now” using a continuous self-report VAS ranging from “no (anxiety/emotion) at all” (0) to “extremely much (anxiety/emotion)” (100).

Change from baseline to 1-month follow-up in physiological parameters during recall of the crucial fear-related memory (M1), will be assessed by bringing up the memory (M1) while the psychophysiological response (heart frequency per minute and blood pressure in mm Hg) will be indexed using an upper arm blood pressure measuring device (CE: 0297) (Table 1).

#### Tertiary outcome measures

The frequency of post-traumatic stress symptoms will be assessed using the *Dutch version of the Impact of Event Scale (IES)* [62]*.* The IES is a 15-item self-report scale, measures the subjective stress following a traumatic event and is one of the most widely used self-report instruments of symptoms of post-traumatic stress [63]. It assesses three PTSD symptom subscales: (1) the intrusion (that is, the loss of voluntary control over the regulation of thoughts) subscale, (2) the avoidance (for example, the extent to which memories are consciously suppressed) subscale, and (3) the hyperarousal subscale. Items (for example, “pictures about it popped into my mind”) are rated on a four-point scale of frequency, ranging from “not at all” (0), “rarely” (1), “sometimes” (3), to “often” (5). The scores are summarized to produce a range of 0 to 75 for the total score. Patients will be explicitly instructed to fill out the IES related to the crucial traumatic memory that exacerbated or initiated their dental anxiety (M1). A score of 26 is considered the cut-off point for a clinically significant level of trauma-related symptomatology [64].

The fear of objects and situations related to the dental setting will be assessed using the *Fear of Stimuli Compromising the Dental Setting (FCDS) questionnaire.* This questionnaire includes the 25 most prevalent stimuli among 67 anxiety-provoking stimuli found in a study in 960 Dutch adults [1]. The fear provoking nature of each item will be scored on a four-point scale, from “not at all fear provoking” (1), to “extremely fear provoking” (4).

During the semi-structured interviews after the first and before the second dental extraction procedure, qualitative aspects of the memory for the recent dental extraction [M2] will be assessed (that is, vividness, emotional charge, avoidance and aversiveness propensity, sense of here and now, the impact of and burden caused by the memory). Patients will be asked to rate these characteristics on standardized continuous self-report VASs, ranging from 0 (for example, “not aversive at all”) to 100 (for example, “extremely aversive”).

The weeks after the dental extractions, the frequency and nature of intrusive thoughts will be indexed using an intrusions diary. Study participants will be asked to record their frequency and nature of their thoughts of the crucial traumatic event that exacerbated or initiated their dental anxiety (M1) for 7 days following the first and second dental extraction procedure.

A number of specific memory characteristics regarding the event that exacerbated or initiated their dental anxiety (M1) will be assessed using a 16-item self-report *Memory Characteristics Questionnaire (MCQ),* on two subscales; re-experiencing (eight items), disorganization (six items), peritraumatic dissociation (one item) and depersonalization and/or derealization during memory retrieval (one item) [65]. All 16 items are rated on a four-point scale ranging from “never” (0) to “all the time” (3).

The patients’ experiences during surgery will be assessed using visual analog scales (ranging from 0 to 100). Directly after the first and second (follow-up) dental extraction procedure, characteristics of participants’ experiences during surgery will be determined. Patients will be asked to rate the propensity of pain experienced during treatment, the degree of experienced reassurance/comforting by their surgeon, the sense of “here and now” when thinking back at this experience and how pleasurable the experience was. Patients will be asked to rate these characteristics on standardized continuous self-report VASs ranging from 0 (for example, “not reassured at all”) to 100 (for example, “completely reassured”).

#### Operative variables

A number of operative variables will be recorded by the surgeon after the first and second dental extraction procedure. These include the nature of the procedure (extraction/surgical removal; numbering of the dental elements extracted using the FDI World Dental Federation/ISO 3950:2009 notation), duration of the surgery, difficulty (that is, whether the surgery was experienced as a “complicated procedure”), number and location of anesthetic capsules administered, observer-rated anxiety (surgeons are asked to rate the question “according to you, how much anxiety did the patient show during treatment” using a continuous observer-rated VAS ranging from (0) “no anxiety at all” to (100) “extremely anxious”), nature of the follow-up treatment, and information on the occurrence of any adverse events.

#### Test of blindness

The patients’ blindness to treatment allocation will be assessed, with respect to the possibility of subsequent biased psychological or physical responses to the intervention. To this end, after all the study medication has been ingested following the first dental extraction, participants will be asked to answer the multiple-choice question “do you think that you have received: (a) the “real” capsules (with propranolol) or (b) the “fake capsules” (with placebo)” with either (a) or (b).

### Data management

To ensure data security and confidentiality, the coded anonymous case report forms will be stored in a locked room to which only the assessors have access.

### Statistical analysis plan

The distributions of demographic variables and quantitative data will be displayed in tables. Associations between categorical variables will be analyzed using the Chi^2^-test. Differences between the propranolol and placebo condition on continuous variables will be analyzed using the independent-samples t-test. Two-way (condition by time) repeated measures ANOVA will be used to compare the propranolol and placebo condition on the dental anxiety reduction between the first and second extraction, possibly with the baseline score as a covariate (if differences exist at baseline). The alpha is set at a significance level of 5 %.

### Ethical approval

This trial (protocol number NL42210.018.13) is being performed in accordance with the Declaration of Helsinki [66] and received ethical approval by the Institutional Review Board “Medisch Ethische Toetsingscommissie Academisch Medisch Centrum” (Medical Ethics Committee of the Academic Medical Center in Amsterdam, the Netherlands) on July 24, 2014. The present study protocol, version 5 (April 1, 2015), has received ethical approval by the latter Institutional Review Board on April 20, 2015. Plans for important protocol modifications will be communicated to the Institutional Review Board, ClinicalTrials.gov and the Netherlands National Trial Register.

### Safety monitoring

In order to (1) protect the rights and well-being of human subjects; (2) ensure that trial data are accurate, complete and verifiable from source documents; and (3) ascertain that the conduct of this trial is in compliance with the currently approved protocol, with Good Clinical Practice (GCP), and with applicable regulatory requirements, this clinical trial is being monitored by a GCP-trained, independent monitor, who is not involved in the clinical trial as part of the trial staff. The monitor will report findings before, during and after the trial.

### Harms

Every adverse event related to the study intervention will be reported on the case report form by the dental surgeon. Participants are insured for harm during and within four years after the end of the trial.

## Discussion

We present a study protocol for a randomized controlled trial to test the effectiveness of 120 mg of perioperative propranolol versus placebo to reduce dental trait anxiety, state anxiety during surgery, as well as physiological responses and memory intrusiveness during recall of a crucial fear-related memory. There is a great need for safe and effective interventions since even specialized dental treatments programs, as applied in dental fear clinics, generally fail to alleviate dental anxiety to a clinically significant degree in a significant proportion of the patients [14].

Dental anxiety has been found to exert a great impact on the quality of daily life because of a vicious cycle of declining psychosocial functioning related to anxiety and embarrassment, and severe pain and deteriorating oral health-related problems due to inadequate dental care [20, 67, 68]. Therefore, targeting the reconsolidation of disturbing memories of aversive experiences in which dental anxiety are rooted, with the purpose of helping to transform these into less disturbing memories, may pave the way to a higher treatment success rate in people with high levels of dental anxiety. This would offer support for routinely prescribing of propranolol in patients who are highly fearful of undergoing a dental extraction.

## Trial status

The trial is ongoing and currently recruiting.

## Abbreviations

AMC: Academic Medical Center of the University of Amsterdam
ANCOVA: analysis of covariance
BP: blood pressure
CRF: case report form
FCDS: fear of stimuli compromising the dental setting
GCP: good clinical practice
GMP: good medical practice
HR: heart rate
ICD-10: 10^th^ revision of the International Classification of Diseases and Related Health Problems
IES-R: Impact of Event Scale - Revised
M1: crucial fear-related memory
M2: memory of recent dental extraction procedure
MCQ: Memory Characteristics Questionnaire
MINI: Mini-International Neuropsychiatric Interview
PTSD: post-traumatic stress disorder
QP: qualified person
S-DAI: Short version of the Dental Anxiety Inventory

## References

[1] Oosterink FMD, de Jongh A, Aartman IHA (2008). What are people afraid of during dental treatment? Anxiety-provoking capacity of 67 stimuli characteristic of the dental setting. Eur J Oral Sci.

[2] Sirin Y, Humphris G, Sencan S, Firat D (2012). What is the most fearful intervention in ambulatory oral surgery? Analysis of an outpatient clinic. Int J Oral Maxillofac Surg.

[3] American Dental Association (2007). The 2005–06 Survey of Dental Services Rendered.

[4] Earl P (1994). Patients’ anxieties with third molar surgery. Br J Oral Maxillofac Surg.

[5] Yusa H, Onizawa K, Hori M, Takeda S, Takeda H, Fukushima S (2004). Anxiety measurements in university students undergoing third molar extraction. Oral Surg Oral Med Oral Pathol Oral Radiol Endod.

[6] de Jongh A, Olff M, van Hoolwerff H, Aartman IHA, Broekman B, Lindauer R (2008). Anxiety and post-traumatic stress symptoms following wisdom tooth removal. Behav Res Ther.

[7] de Jongh A, van Wijk AJ, Lindeboom JA (2011). Psychological Impact of Third Molar Surgery: A 1-Month Prospective Study. J Oral Maxillofac Surg.

[8] Lago-Méndez L, Diniz-Freitas M, Senra-Rivera C, Seoane-Pesqueira G, Gándara-Rey JM, García-García A (2009). Postoperative recovery after removal of a lower third molar: role of trait and dental anxiety. Oral Surg Oral Med Oral Pathol Oral Radiol Endod.

[9] Aznar-Arasa L, Figueiredo R, Valmaseda-Castellón E, Gay-Escoda C (2014). Patient anxiety and surgical difficulty in impacted lower third molar extractions: a prospective cohort study. Int J Oral Maxillofac Surg.

[10] Lamont T, Matthew L, Cousins D, Green J (2009). Avoiding midazolam overdose: summary of a safety report from the National Patient Safety Agency. BMJ.

[11] van Minnen A, Arntz A, Keijsers GPJ (2002). Prolonged exposure in patients with chronic PTSD: predictors of treatment outcome and dropout. Behav Res Ther.

[12] Wide Boman U, Carlsson V, Westin M, Hakeberg M (2013). Psychological treatment of dental anxiety among adults: a systematic review. Eur J Oral Sci.

[13] Gordon D, Heimberg RG, Tellez M, Ismail AI (2013). A critical review of approaches to the treatment of dental anxiety in adults. J Anxiety Disord.

[14] Aartman I, Jongh A (2000). Dental anxiety reduction and dental attendance after treatment in a dental fear clinic: a follow‐up study. Community Dent Oral Epidemiol.

[15] de Jongh A, Aartman IHA, Brand N (2003). Trauma-related phenomena in anxious dental patients. Community Dent Oral Epidemiol.

[16] De Jongh A, Fransen J, Oosterink-Wubbe F, Aartman I (2006). Psychological trauma exposure and trauma symptoms among individuals with high and low levels of dental anxiety. Eur J Oral Sci.

[17] Oosterink FMD, de Jongh A, Aartman IHA (2009). Negative events and their potential risk of precipitating pathological forms of dental anxiety. J Anxiety Disord.

[18] de Jongh A, Muris P, ter Horst G, Duyx MP (1995). Acquisition and maintenance of dental anxiety: the role of conditioning experiences and cognitive factors. Behav Res Ther.

[19] van Houtem CMHH, van Wijk AJ, de Jongh A (2015). Presence, Content, and Characteristics of Memories of Individuals with Dental Phobia. Appl Cogn Psychol.

[20] Berggren U (1993). Psychosocial effects associated with dental fear in adult dental patients with avoidance behaviours. Psychol Health.

[21] Black JW, Crowther AF, Shanks RG, Smith LH, Dornhorst AC (1964). A new adrenergic betareceptor antagonist. Lancet.

[22] Webb AJS, Fischer U, Mehta Z, Rothwell PM (2010). Effects of antihypertensive-drug class on interindividual variation in blood pressure and risk of stroke: a systematic review and meta-analysis. Lancet.

[23] Freemantle N, Cleland J, Young P, Mason J, Harrison J (1999). beta Blockade after myocardial infarction: systematic review and meta regression analysis. BMJ.

[24] Fuster V, Rydén LE, Cannom DS, Crijns HJ, Curtis AB, Ellenbogen KA (2006). ACC/AHA/ESC 2006 Guidelines for the Management of Patients with Atrial Fibrillation: a report of the American College of Cardiology/American Heart Association Task Force on Practice Guidelines and the European Society of Cardiology Committee for Practice. Circulation.

[25] Wheatley D (1969). Comparative effects of propranolol and chlordiazepoxide in anxiety states. Br J Psychiatry.

[26] Kathol RG, Noyes R, Slymen DJ, Crowe RR, Clancy J, Kerber RE (1980). Propranolol in chronic anxiety disorders. A controlled study. Arch Gen Psychiatry.

[27] Meibach RC, Dunner D, Wilson LG, Ishiki D, Dager SR (1987). Comparative efficacy of propranolol, chlordiazepoxide, and placebo in the treatment of anxiety: a double-blind trial. J Clin Psychiatry.

[28] Grosz HJ (1972). Narcotic withdrawal symptoms in heroin users treated with propranolol. Lancet.

[29] Yorkston NJ, Zaki SA, Malik MK, Morrison RC, Havard CW (1974). Propranolol in the control of schizophrenic symptoms. Br Med J.

[30] Ratey JJ, Bemporad J, Sorgi P, Bick P, Polakoff S, O’Driscoll G (1987). Open trial effects of beta-blockers on speech and social behaviors in 8 autistic adults. J Autism Dev Disord.

[31] Fleminger S, Greenwood RJ, Oliver DL (2006). Pharmacological management for agitation and aggression in people with acquired brain injury. Cochrane Database Syst Rev.

[32] Brewer C (1972). Beneficial effect of beta-adrenergic blockade on “exam. nerves”. Lancet.

[33] Stone W, Gleser G, Gottschalk L (1973). Anxiety and beta-adrenergic blockade. Arch Gen Psychiatry.

[34] Drew PJ, Barnes JN, Evans SJ (1985). The effect of acute beta-adrenoceptor blockade on examination performance. Br J Clin Pharmacol.

[35] Brantigan CO, Brantigan TA, Joseph N (1982). Effect of beta blockade and beta stimulation on stage fright. Am J Med.

[36] Clark DB, Agras WS (1991). The assessment and treatment of performance anxiety in musicians. Am J Psychiatry.

[37] Elman MJ, Sugar J, Fiscella R, Deutsch TA, Noth J, Nyberg M (1998). The effect of propranolol versus placebo on resident surgical performance. Trans Am Ophthalmol Soc.

[38] Dyck JB, Chung F (1991). A comparison of propranolol and diazepam for preoperative anxiolysis. Can J Anaesth.

[39] Jakobsson J, Rane K, Ryberg G (1995). Oral premedication one hour before minor gynaecological surgery--does it have any effect? A comparison between ketobemidone, lorazepam, propranolol and placebo. Acta Anaesthesiol Scand.

[40] Mealy K, Ngeh N, Gillen P, Fitzpatrick G, Keane FB, Tanner A (1996). Propranolol reduces the anxiety associated with day case surgery. Eur J Surg.

[41] Liu HH, Milgrom P, Fiset L (1991). Effect of a beta-adrenergic blocking agent on dental anxiety. J Dent Res.

[42] Sevenster D, Beckers T, Kindt M (2013). Prediction error governs pharmacologically induced amnesia for learned fear. Science.

[43] Kindt M, Soeter M, Vervliet B (2009). Beyond extinction: erasing human fear responses and preventing the return of fear. Nat Neurosci.

[44] Debiec J, Ledoux JE (2004). Disruption of reconsolidation but not consolidation of auditory fear conditioning by noradrenergic blockade in the amygdala. Neuroscience.

[45] Finnie PSB, Nader K (2012). The role of metaplasticity mechanisms in regulating memory destabilization and reconsolidation. Neurosci Biobehav Rev.

[46] Nader K, Schafe GE, Le Doux JE (2000). Fear memories require protein synthesis in the amygdala for reconsolidation after retrieval. Nature.

[47] Merlo E, Milton AL, Everitt BJ (2015). Enhancing cognition by affecting memory reconsolidation. Curr Opin Behav Sci.

[48] Merlo E, Milton AL, Goozee ZY, Theobald DE, Everitt BJ (2014). Reconsolidation and Extinction Are Dissociable and Mutually Exclusive Processes: Behavioral and Molecular Evidence. J Neurosci.

[49] Soeter M, Kindt M (2010). Dissociating response systems: erasing fear from memory. Neurobiol Learn Mem.

[50] Johansen JP, Cain CK, Ostroff LE, LeDoux JE (2011). Molecular mechanisms of fear learning and memory. Cell.

[51] Lonergan MH, Olivera-Figueroa LA, Pitman RK, Brunet A (2013). Propranolol’s effects on the consolidation and reconsolidation of long-term emotional memory in healthy participants: a meta-analysis. J Psychiatry Neurosci.

[52] Brunet A, Orr SP, Tremblay J, Robertson K, Nader K, Pitman RK (2008). Effect of post-retrieval propranolol on psychophysiologic responding during subsequent script-driven traumatic imagery in post-traumatic stress disorder. J Psychiatr Res.

[53] American Psychiatric Association (2000). Diagnostic and Statistical Manual of Mental Disorders, Fourth Edition, Text Revision.

[54] Oosterink FMD, de Jongh A, Hoogstraten J (2009). Prevalence of dental fear and phobia relative to other fear and phobia subtypes. Eur J Oral Sci.

[55] American Psychiatric Association (2013). Diagnostic and Statistical Manual of Mental Disorders.

[56] Sheehan DV, Lecrubier Y, Sheehan KH, Amorim P, Janavs J, Weiller E (1998). The Mini-International Neuropsychiatric Interview (M.I.N.I.): the development and validation of a structured diagnostic psychiatric interview for DSM-IV and ICD-10. J Clin Psychiatry.

[57] Reynolds M, Brewin CR (1999). Intrusive memories in depression and posttraumatic stress disorder. Behav Res Ther.

[58] Pitman RK, Sanders KM, Zusman RM, Healy AR, Cheema F, Lasko NB (2002). Pilot study of secondary prevention of posttraumatic stress disorder with propranolol. Biol Psychiatry.

[59] McGaugh JL (2000). Memory - a century of consolidation. Science.

[60] Aartman IH (1998). Reliability and validity of the short version of the Dental Anxiety Inventory. Community Dent Oral Epidemiol.

[61] van Wijk AJ, Makkes PC (2008). Highly anxious dental patients report more pain during dental injections. Br Dent J.

[62] Horowitz M, Wilner N, Alvarez W (1979). Impact of Event Scale: a measure of subjective stress. Psychosom Med.

[63] Joseph S (2000). Psychometric evaluation of Horowitz’s Impact of Event Scale: a review. J Trauma Stress.

[64] Kleber R, Brom D, Defares P (1992). Coping with Trauma: Theory, Prevention and Treatment.

[65] Hagenaars MA, van Minnen A, Hoogduin CAL, Verbraak M (2009). A transdiagnostic comparison of trauma and panic memories in PTSD, panic disorder, and healthy controls. J Behav Ther Exp Psychiatry.

[66] World Medical Association (2013). World Medical Association Declaration of Helsinki: ethical principles for medical research involving human subjects. JAMA.

[67] De Jongh A, Schutjes M, Aartman IHA (2011). A test of Berggren’s model of dental fear and anxiety. Eur J Oral Sci.

[68] Armfield JM, Stewart JF, Spencer AJ (2007). The vicious cycle of dental fear: exploring the interplay between oral health, service utilization and dental fear. BMC Oral Health.

